# Avian pathogenic *Escherichia coli*: Epidemiology, virulence and pathogenesis, diagnosis, pathophysiology, transmission, vaccination, and control

**DOI:** 10.14202/vetworld.2024.2747-2762

**Published:** 2024-12-06

**Authors:** Aswin Rafif Khairullah, Daniah Ashri Afnani, Katty Hendriana Priscilia Riwu, Agus Widodo, Sheila Marty Yanestria, Ikechukwu Benjamin Moses, Mustofa Helmi Effendi, Sancaka Chasyer Ramandinianto, Syahputra Wibowo, Ima Fauziah, Muhammad Khaliim Jati Kusala, Kartika Afrida Fauzia, Abdul Hadi Furqoni, Ricadonna Raissa

**Affiliations:** 1 Research Center for Veterinary Science, National Research and Innovation Agency (BRIN), Jl. Raya Bogor, Km. 46 Cibinong, Bogor, West Java, Indonesia; 2 Department of Microbiology and Parasitology, Faculty of Veterinary Medicine, Universitas Pendidikan Mandalika, Jl. Pemuda No. 59A, Dasan Agung Baru, Mataram, West Nusa Tenggara, Indonesia; 3 Department of Veterinary Public Health, Faculty of Veterinary Medicine, Universitas Pendidikan Mandalika. Jl. Pemuda No. 59A, Dasan Agung Baru, Mataram 83125, West Nusa Tenggara, Indonesia; 4 Department of Health, Faculty of Vocational Studies, Universitas Airlangga, Jl. Dharmawangsa Dalam Selatan, No. 28-30, Kampus B Airlangga, Surabaya, East Java, Indonesia; 5 Laboratory of Veterinary Public Health, Faculty of Veterinary Medicine, Universitas Wijaya Kusuma Surabaya, Jl. Dukuh Kupang XXV No.54, Dukuh Kupang, Dukuh Pakis, Surabaya, East Java, Indonesia; 6 Department of Applied Microbiology, Faculty of Science, Ebonyi State University, Abakaliki, Nigeria; 7 Division of Veterinary Public Health, Faculty of Veterinary Medicine, Universitas Airlangga, Jl. Dr. Ir. H. Soekarno, Kampus C Mulyorejo, Surabaya, East Java, Indonesia; 8 Lingkar Satwa Animal Care Clinic. Jl. Sumatera No. 31L, Gubeng, Surabaya, East Java, Indonesia; 9 Eijkman Research Center for Molecular Biology, National Research and Innovation Agency (BRIN), Jl. Raya Bogor, Km. 46 Cibinong, Bogor, West Java, Indonesia; 10 Research Center for Preclinical and Clinical Medicine, National Research and Innovation Agency (BRIN), Jl. Raya Bogor, Km. 46 Cibinong, Bogor, West Java, Indonesia; 11 Department of Environmental and Preventive Medicine, Faculty of Medicine, Oita University, 700 Dannoharu, Oita, Japan; 12 Center for Biomedical Research, National Research and Innovation Agency (BRIN), Jl. Raya Bogor, Km. 46 Cibinong, Bogor, West Java, Indonesia; 13 Department of Pharmacology, Faculty of Veterinary Medicine, Universitas Brawijaya, Jl. Veteran No.10-11, Ketawanggede, Lowokwaru, Malang, Indonesia

**Keywords:** avian pathogenic *Escherichia coli*, colibacillosis, *Escherichia coli*, poultry, public health

## Abstract

Avian pathogenic *Escherichia coli* (APEC) causes colibacillosis in poultry; this type of bacteria is an extraintestinal pathogen *E. coli*. Unlike other *E. coli* pathogen groups, the characteristics of APECs cannot be identified by a single group. Serotyping and biotyping are frequently performed for isolates found in colibacillosis infections. The establishment, transmission, and persistence of this pathogenic strain in chicken populations are determined by the intricate interactions of multiple elements that make up the epidemiology of APEC. APEC employs many virulence and pathogenesis factors or mechanisms to infect chickens with colibacillosis. These factors include invasives, protectins, adhesins, iron acquisition, and toxins. In addition, the pathogenicity of APEC strains can be evaluated in 2–4 *week*-old chicks. The impact of unfavorable environmental conditions has also been documented, despite direct contact being demonstrated to be a significant element in transmission in APEC. Chickens are immunized against colibacillosis using a variety of vaccines. Nevertheless, commercially available vaccinations do not offer sufficient immunity to protect birds from APEC strains. Hatching egg contamination is one of the main ways that APECs spread throughout chicken flocks. Farmers also need to be mindful of storing discarded materials near the manure-watering area, removing them when necessary, and replacing wet materials with dry materials when needed. This review aimed to explain the characteristics, epidemiology, virulence, pathogenesis, diagnosis, pathophysiology, transmission, vaccination, and control of APEC.

## Introduction

The well-characterized Gram-negative bacterium *Escherichia coli* is present in upper respiratory and digestive tracts of poultry and mammals [1]. Conversely, a possible risk factor for colibacillosis in poultry is the existence of pathogenic *E. coli* in the environment, digestive tract, or respiratory system [2]. Avian pathogenic *E. coli* (APEC) causes colibacillosis in poultry; this type of bacteria is an extraintestinal pathogen *E. coli* (ExPEC) [3]. These conditions indicate colibacillosis, cellulitis, omphalitis, pericarditis, perihepatitis, and salpingitis [4]. The environment and the host’s stress levels determine disease transmission; organs can still be infected, and colibacillosis can result from less pathogenic strains with fewer virulence genes [5].

APEC is common in Indonesia, and several studies have demonstrated this. The prevalence of APEC in quail was 32.26% [6]. Putri *et al*. [7] reported the prevalence of APEC in broiler chickens at 40%, but Effendi *et al*. [8] reported a higher prevalence of 73.5%. APEC causes quite high economic losses in Indonesia and results in losses of 13.10% of total poultry assets both directly (weight loss, decreased egg production, and increased total mortality) and indirectly (cleaning, disinfection, and labor compensation) if the disease occurs [9].

The numerous virulence characteristics of APEC bacterial strains enable the bacteria to leave the digestive tract and travel to different interior organs, where they can cause illness [9]. High mortality rates, weight loss, decreased egg production, and rotting of poultry carcasses during slaughter and storage are all potential financial losses associated with APEC infections in the worldwide poultry sector [10]. Human foodborne illnesses can be caused by poultry meat. Poultry producers, suppliers, consumers, and public health officials worldwide continue to be concerned about pathogenic *E. coli*, including APEC and other spoilage microbes in poultry [2]. *E. coli* is a common bacterium linked to foodborne diseases in most countries worldwide [11].

Antibiotic use for illness treatment and prevention has increased due to the prevalence of colibacillosis and other diseases in poultry [12]. It is anticipated that this will lead to the development of antibiotic resistance, also known as antimicrobial resistance (AMR), in the chicken farming industry [13]. Antibiotic resistance in APEC is a global public health concern that must be addressed by all government and social sectors. In poultry farming, uncontrolled antibiotic use can lead to antibiotic resistance [14]. Farmers believe that using antibiotics to prevent disease is a low-cost and side-effects-free endeavor, which accounts for the widespread usage of antibiotics without a prescription from a licensed veterinarian [15].

**Figure-1:**
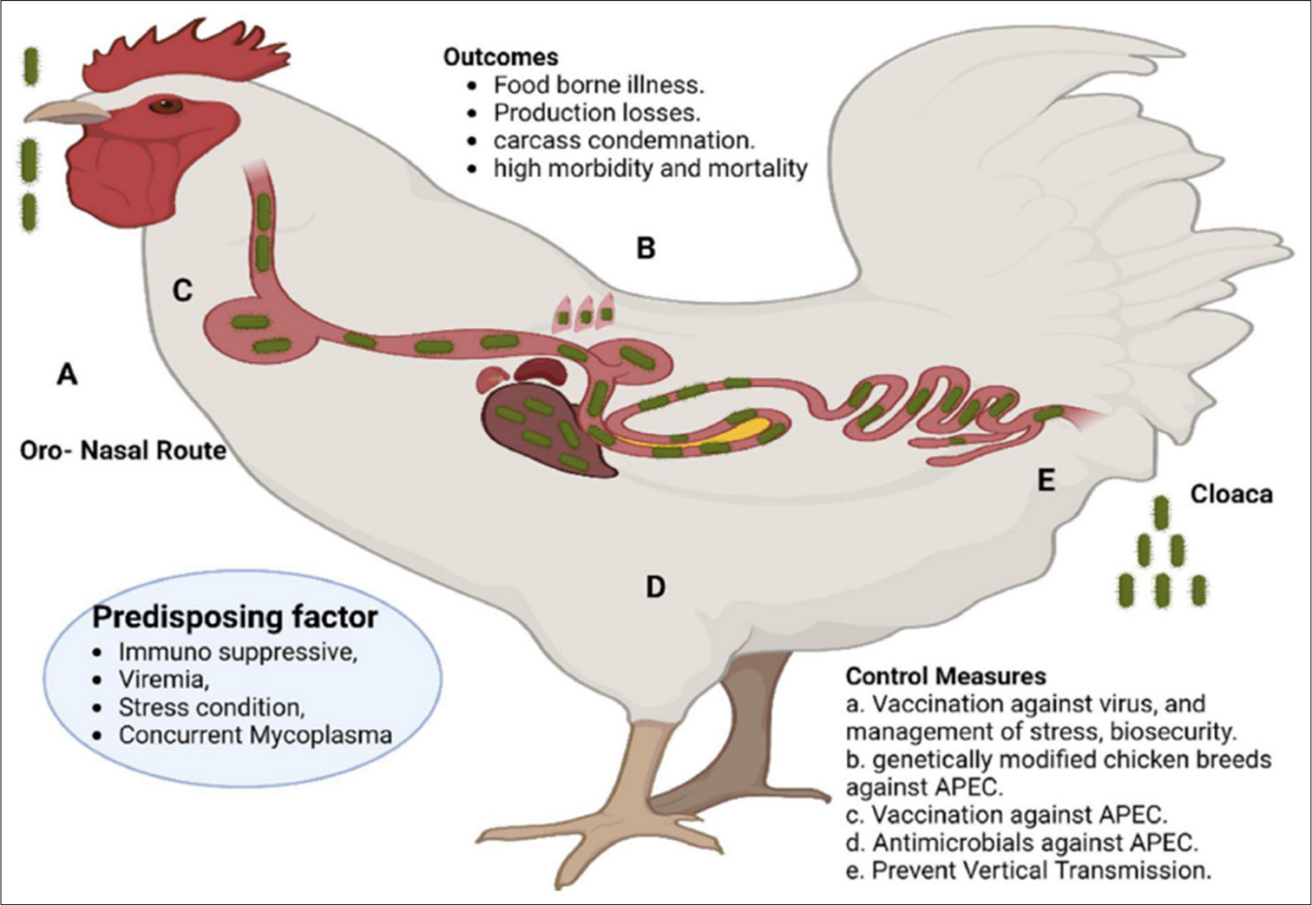
A schematic representation of avian pathogenic *Escherichia coli* infection in chickens after entering through oral, nasal, or cloacal routes [17].

APEC transmission in chickens can be increased by several factors, including unfavorable conditions in poultry houses, high levels of fecal dust and ammonia contamination, subpar or ineffective farming practices, contaminated water and feed, stress, underlying viral diseases, contaminated eggs, insect vectors, cannibalism, and proximity to other animals in poultry farming [16]. The fecal-oral, respiratory, and vaginal pathways through the cloaca are the primary entrance points for APEC (Figure-1) [17]. The most researched forms of colibacillosis are respiratory and fecal-oral. Nevertheless, because cloacal infection causes outbreaks of salpingitis-peritonitis syndrome, it is equally crucial to investigate this type of infection [18]. Furthermore, infection by antibiotic-resistant strains of APEC will make management extremely challenging.

Raising public awareness of the risk of APEC transmission in poultry farming and the possible harm to public health can decrease and prevent colibacillosis in poultry flocks. This review aims to comprehensively explain APEC as the main causative agent of colibacillosis, starting with its characteristics, epidemiology, virulence, pathogenesis, diagnosis, pathophysiology, transmission, vaccination, and control.

## Characteristics

Unlike other *E. coli* pathogen groups, the characteristics of APECs cannot be identified by a single group. The following summarizes a few phenotypic and genotypic traits connected to this class of diseases [19].

Serotyping and biotyping are frequently performed for isolates found in colibacillosis infections. The predominant serogroups of *E. coli* recovered from sick birds are O1, O2, and O78 [19]. Thus, representative serotype strains offer a focal point for deciphering the mechanisms of APEC pathogenicity and for creating and assessing potential vaccines. Given that this dominant serogroup may also be recovered from the feces of birds that appear to be in good health, the hypothesis that the digestive tract may serve as a substantial natural reservoir for APEC and that predisposing factors may be necessary to produce the disease is supported [20]. Many investigations have revealed similarities between commensal *E. coli* and APEC, including serogroup, suggesting that APEC developed from commensal *E. coli* after gaining pathogenic qualities [21–23]. The various features observed include the outer membrane protein profile, pathogenicity profile, and multilocus enzyme electrophoresis within a serotype. To define APEC isolates, tests for motility, hemolysis, lactose fermentation, complement resistance, serotyping, aerobactin and colicin V synthesis, and embryo lethality are occasionally performed, although phenotypes can vary [24].

In APECs, plasmid-mediated horizontal gene transfer induces a great deal of variability. Several potential virulence factors are carried by APEC plasmids, yet some of these factors were discovered in isolates of *E. coli* from chickens that appeared to be in good condition [8, 25]. There is a significant variation in amount, size, and virulence traits of plasmids in both APECs and isolates from healthy chickens [26]. However, other studies did not identify colicin-related genes or APEC-specific plasmid replicons in commensal bacteria [27]. There is little question that the abundance of virulence genes carried by APEC plasmids has contributed to the present classification of APEC genotypes. APEC is more likely than avian fecal *E. coli* to carry genes encoding iron uptake and transport mechanisms, which are located in a 94 kb area of the 180 kb ColV plasmid [28]. Numerous studies have demonstrated a connection between ColV plasmids and APEC pathogenicity [3, 9, 29].

The chicken trachea becomes more colonized when an avirulent *E. coli* strain is transformed with a recombinant plasmid (pHK11) expressing colicin V [30]. Recently, a commensal *E. coli* strain was conjugated with two APEC plasmids (large R and ColV), which improved its ability to colonize rodent kidneys, grow in human urine, and destroy chicken eggs [23]. A pair of multiplex polymerase chain reaction (PCR) methods has been recently developed to identify APECs using a set of common characteristics, such as plasmid-borne phenotypes. Strains from sick birds were classified as APEC if they contained at least four of the eight genes included in the study: Iron repressible protein *(irp*2), enteroaggregative toxin *(ast*A), temperature-sensitive hemagglutinin *(ts*h), P fimbriae *(pap*C), aerobactin *(iuc*D), increased serum survival protein *(iss*), colicin plasmid operon genes *(cva/cvi*), and vacuolating autotransporter protein (vat), whereas nonpathogenic isolates had none or at most three genes [31].

Four multiplex PCRs were recently developed for virulence genotyping to determine the evolutionary links between ExPEC strains from birds and humans [32]. However, surprisingly, little is known about the gene combinations that are critical for causing systemic infections with APEC. Nonetheless, prior research has demonstrated that Tsh/Pap/Iuc pathotypes, Tsh/Pil/Iuc, and the virulence-related markers. Tsh and Iuc are crucial for APEC [33]. The relationship between APEC strain serogroup and virulence gene patterns is not very strong. The link between several virulence genes may reflect the existence of distinct subpathotypes or pathotypes within the current APEC group, indicating that the potential subpathotypes may use distinct virulence mechanisms [34]. It is clear that more revisions to the APEC concept are required.

## Epidemiology

The establishment, transmission, and persistence of this pathogenic strain in chicken populations are determined by the intricate interactions of multiple elements that constitute the epidemiology of APEC. The epidemiology of APEC includes the crucial component of avian host vulnerability [9]. Some factors affecting the risk of infection include age, immune status, and general health [2]. Although this disease affects all age groups, chicks and stressed birds are especially susceptible to this infection [35]. The environment is crucial for the transmission of APEC. In chicken farming, APEC’s survivability is related to tainted water sources, overcrowding, and inadequate sanitation [36]. Thus, robust biosecurity measures are required to mitigate environmental risk factors.

Another component of its epidemiology is the genetic diversity of the APEC strains. A genomic study by Desvaux *et al*. [37] has identified numerous virulence factors and transportable genetic components that support APEC’s adaptability and evolution. The significance of continuous surveillance in tracking strain fluctuation was demonstrated by the observed genetic diversity. One important aspect of APEC epidemiology is the horizontal gene transfer across bacteria [38]. A portion of the virulence and antibiotic resistance genes that are transferred among APEC strains can be attributed to cellular genetic factors such as bacteriophages and plasmids [39]. Recognizing these pathways is essential for anticipating and managing APEC outbreaks.

Ewers *et al*. [40] have contributed to our understanding of the molecular epidemiology of APEC in chicken farming. They also discussed the significance of monitoring genetic alterations and the effects of horizontal gene transfer. Variations in the genetic makeup of the bacteria, environmental factors, and host characteristics complicate the epidemiology of APEC. Wibisono *et al*. [9] demonstrated the importance of continuing monitoring and conducting research to develop efficient management and preventative strategies that safeguard chicken health.

## Virulence and pathogenesis

APEC employs many virulence and pathogenesis factors and mechanisms to infect chickens with colibacillosis. These include poisons, iron acquisition mechanisms, invasives, protectins, and adhesins. These factors include attachment to host cells, invasion of host cells, survival within phagocytic cells (macrophages), colonization of tissue, bloodstream persistence, cellular proliferation or replication, lysis, and cell damage, sequestering metals from bodily fluids for growth, resistance to oxidative and environmental stress, serum bactericidal activity, motility, and biofilm formation.

### Adhesin

Adhesins are bacterial cell adhesions or surface elements that help bacteria adhere to other surfaces or cells, usually the host in which they live or infect [41]. Adherence is a crucial stage in the pathogenesis or infection of bacteria and is necessary for the organism to colonize a new host [42]. The main factors promoting adherence to APEC are type 1 fimbriae, S fimbriae, and P fimbriae [43]. Some of the genes encoding these fimbriae and additional adhesins, *fim*C (type 1 fimbriae), *fim*H, *pap*A, *pap*EF, *sfa*/*sfa*S (S fimbriae), *crl*, *pap*GI, *pap*C, *pap*GII, *flg*E (flagellar hook), *pap*GIII, *fel*A (P fimbriae), *foc*GE (F1C fimbriae), *lpf*A, *afa*IBC (afimbriae), *lpf*0154 (long polar fimbriae), *lpf*0141, *mat*/*ecp*A (fimbrillin), *csg* (curli), *yqi*G (putative outer membrane messenger protein), *bma*E (M hemagglutinin), *iha* (adhesin homolog IrgA), *hra*/*hrl*A/*hek* (heat-stable agglutinin), *tsh* (temperature-sensitive hemagglutinin), and *kii* (capsule-encoding K gene) have been reported to occur in APEC [16]. These adhesins regulate macrophage APEC motility, biofilm formation, and survival [44]. In addition, colonization, adhesion, and resistance to environmental stress are facilitated by the fimbriae-encoding gene *yfc*O, whereas adhesion, intracellular survival, and motility are improved by *yad*C [45]. Adhesion, colonization, and biofilm formation are also facilitated by the adhesin autotransporter genes (*aat*A, *aat*B, and *upa*B) [46]. Several more genes (*fde*C, *fdt*A, *yjh*B, *rlu*D, and *ecp*R) were discovered to be in charge of adhesion in chicken and human cell lines by screening random transposon mutants [47].

### Invasin

A class of proteins known as invasin is linked to the entry of infectious cells into host cells. Early in infection, invasiveness contributes to the admission of bacteria [48]. Several genes encoding the invasives, *tia* (toxigenic invasion locus), *ibe*A (also called *ibe*10), *ibe*B (invasion protein), and *gim*B (genetic island associated with neonatal meningitis), have been reported in APEC isolates [49]. Furthermore, invasions help APEC survive oxidative damage, colonization, biofilm formation, and proliferation in the host that is brought on by macrophages [9]. The *ibe*R operon regulator plays a role in invasion, resistance to environmental stressors and serum, and the production of virulence genes [50]. Similarly, the suspected invasive gene *ych*O contributes to biofilm formation, motility, adhesion, invasion, and the production of metabolic and membrane proteins [51].

### Iron acquisition system

Once bacteria have successfully colonized or attacked their host, iron is a crucial micronutrient for proliferating and multiplying in the host [1]. To absorb iron from bodily fluids, APEC uses a distinct iron acquisition system that consists of transporters and many siderophores, including yersiniabactin, salmochelin, and aerobactin [52]. Several genes that encode for iron uptake and transport systems, *mnt*H (iron and manganese transporter), *aer*J (aerobactin), *iut*A, *sit*ABCD, *fyu*A (yersiniabactin), *feo*B (iron ion transporter), *irp*2 (iron repressor protein), *iro*BCDEN (salmochelin), *fep*C (ferric enterobactin transporter), *eit*ABCD (putative iron transporter), *ire*A (iron-regulated virulence gene), *chu*A (outer membrane hemin receptor), *iuc*CD, and *bfr* (bacterioferritin) have been reported in APEC [53]. These siderophores and transporters also mediate the expression of other virulence genes, invasion, resistance to environmental stress, colonization, and persistence of APEC in host cells [54]. Moreover, genes encoding the outer membrane efflux protein (*tol*C), enterobactin production, and transport genes (*ent*E and *ent*S) work together to promote invasion, colonization, and persistence [55].

### Protectin

Bacteria are shielded by protectin from the host’s immune system and other harmful environments [56]. In particular, protection offers defense against complement-mediated bactericidal effects in human serum and phagocytic engulfment by macrophages. The lipopolysaccharide (LPS) comprises bacterial capsules, LPS components, and outer membrane proteins [57]. Several genes that encode for protection, *tra*T (complement resistance protein), *kfi*C-K5 (glycosyl transferase), *iss* (increased serum survival), *kps*MT(II), *omp*T (outer membrane protease), *kps*MT(III), *kps*MT(K1), *neu*S, *neu*C, *neu*D (capsule), and *bet*A (choline dehydrogenase), have been reported in APEC [58]. In addition to shielding APECs from host defenses, this protection facilitates APEC adhesion, intracellular survival, colonization, invasion, and proliferation on the host [59].

In addition, the outer membrane proteins PagP and YbjX contribute to intracellular survival, invasion resistance, and resistance to environmental and serum stress [60]. In a similar vein, OmpA, another protein found on the outer membrane, also helps macrophages survive APEC [61]. LPS production genes, *wzy* (O-antigen polymerase), and *waa*L (O-antigen ligase) genes, aid in adhesion, colonization, motility, invasion, and biofilm formation, as well as intracellular survival and resistance to phagocytosis and environmental stress [62]. Similarly, the lipid A biosynthesis gene *lpx*M (myristoyl transferase) has been implicated in invasion, intracellular survival, colonization, cytokine gene expression control and nitric oxide generation [63]. In the meantime, SOD (superoxide dismutase) encourages the production of biofilms and shields APEC from host responses caused by reactive oxygen species [64].

### Toxins

Toxins are biological poisons that render germs more capable of attacking and damaging tissues [65]. Several genes encode for various types of toxins: *ast*A, *hly*E (putative avian hemolysin), *hly*A, vat (vacuolating autotransporter toxin), *cdt*B, *hly*F, *stx*2f (Shiga toxin variant), *cdt*S (cytolethal distending factor), sat (secret autotransporter toxin), *esp*C (serine protease), EAST-1 (heat-stable enterotoxin), *pic* (serine protease autotransporter), and *ace*4/35 (acetylcholine esterase) have been reported in APEC [66]. In addition, this toxin promotes the development of outer membrane vesicles, agglutination, motility, colonization, biofilm formation, and vacuolization [67].

## Diagnosis

### Pathogenicity of one-day-old chicks

The most reliable method for identifying virulence in an *E. coli* strain is to perform a pathogenicity test on one-day-old chicks [25]. The virulence of this strain was assessed using the APEC subcutaneous inoculation method on one-day-old embryos or chicks, based on a 50% fatal dose [68]. This technique can be used to isolate APEC from infected chicks.

In addition, the pathogenicity of APEC strains can be evaluated in 2–4-week-old chicks [69]. This technique allows the illness to spread while it is in the field. This approach involves inoculating the sample into the nasopharynx or trachea following an initial challenge with a different agent [67]. This is because *E. coli* is typically caused by secondary infections caused by mycoplasma, infectious bronchitis virus, Newcastle disease, or environmental factors [70–72]. The typical clinical manifestations of colibacillosis include internal organ contamination, hemorrhage, pericarditis, perihepatitis, fibrin in the air sacs, and weight loss [18]. This model verified that the injected strain was pathogenic.

In this scenario, *E. coli* strains immediately injected into air sacs are considered high-performance models because the triggering agent does not need to be pre-treated [73]. Compared with inoculating the upper respiratory tract, inoculating the air sac permits more uniform lesions typical of colibacillosis [74].

### Isolation and biochemistry

Most microorganisms require culture media to grow *in vitro*. Some of these mediums are used to support the development of microorganisms and distinguish one sample from another according to its biochemical properties. Furthermore, because they are used to distinguish closely related organisms, selective culture medium permits the growth of certain diseases and prevents the growth of other diseases.

Levine-modified eosin-methylene blue agar, a differential medium first discovered by Holt-Harris and Teague in 1916, is used to identify and isolate Gram-negative intestinal infections. In organisms that ferment lactose and those that do not, dyes, eosin Y, and methylene blue are used as markers of differentiation in response to lactose and saccharose fermentation [75].

MacConkey agar (MCA) is a selective medium containing bile salts and crystal violet, which prevent the growth of Gram-positive bacteria, particularly enterococci and staphylococci [76]. Gram-negative bacteria grow more effectively with MCA. Furthermore, MCA is frequently used to separate *Salmonella* Typhi from the coliform group as well as to identify and isolate all strains of typhoid, paratyphoid, and dysentery [77].

The isolation of *E. coli* in Sorbitol MCA (SMCA) and SMCA plus cefixime and tellurite media is widely used [78]. This medium can potentially provide false-positive results for bacteria that do not ferment sorbitol, including *Hafnia alvei*, *Morganella morganii*, *Proteus* spp., *Providencia* spp., and *Aeromonas* spp. [79].

*E. coli*’s primary biochemical trait is its ability to ferment maltose, rhamnose, mannitol, xylose, glycerol, glucose, arabinose, mannose, and sorbitol to produce acids and gases. There are differences in the applications of additional sugars such as dulcitol, ornithine, saccharose, adonitol, salicin, raffinose, and arginine [80]. Positive results were observed for the synthesis of lysine, indole, and motility. Tests for oxidase, citrate utilization, urea hydrolysis, gelatin melting, and H_2_S (hydrogen sulfide) production are likely to yield negative results [81]. Other tests like Voges Proskauer and methyl red are anticipated to yield positive and negative results, respectively.

The ability of *E. coli* to ferment glucose by releasing gas and acid is its primary trait [82]. The β-galactose enzyme converts lactose into glucose and galactose [83]. This enzyme is used to distinguish *E. coli* from *Shigella* and *Salmonella* species.

### Serotype

Within the serogroup, APEC strains are categorized as serotype O: K:H [84]. Somatic antigens belong to serogroup O, capsular antigen to K1, flagella antigens to serogroup H, and antigens type 1 (F1A), P (F11), and curli fimbriae related to fimbriae [9]. APEC strains include 177 “O” antigens, 100 “K” antigens, and 56 “H” flagellar antigens [59]. In contrast, a different study found that APEC has 167 “O,” 74 “K,” 53 “H,” and 17 fimbriae antigens [23].

Somatic “O” antigen is an endotoxin secreted during the lysis of *E. coli* and is largely composed of polysaccharides [82]. The O serogroups involved in avian colibacillosis are O1, O2, O3, O4, O6, O8, O11, O15, O18, O21, O35, O36, O50, O64, O71, O74, O75, O78, O87, O88, O95, O100, O103, O109, O115, O119, O132, O141, and O152 [85]. APEC’s most prevalent serogroups are O1, O2, O8, O35, and O78 [12].

The adherence of *E. coli* to the cell surface is linked to fimbrial antigen, also known as the “F” antigen [1]. Fimbriae, also known as pili, are associated with the presence of this carbohydrate in cells and exhibit sensitivity or resistance to mannose agglutination [86, 87]. Most APECs have type 1 and curli fimbriae [88]. Curli, type 1 and P fimbriae APEC may contain F1C, which clings to buccal epithelial cells; Dr fimbriae, which persist in kidney tissue; and type 1-like fimbriae [89]. Avian *E. coli* 1 fimbriae (AC/1 fimbriae) are a novel fimbrial group of the fimbrial adhesin family [22].

Another antigen that codes for *E. coli* is flagellin, which is sometimes known as the “H” antigen [90]. The majority of APECs include flagellar antigens, which indicate APEC motility. Flagella offers intestinal mucus penetration, invasion, colonization, and persistence in a specific-pathogen-free chicken model [91]. The virulence of *E. coli* is not linked to this antigen.

The major APEC serotype contains the polysaccharide polysialic acid, which comprises the K capsule antigen [92]. This is related to virulence. There is a connection between this antigen and extraintestinal infections. In the avian isolate O2:K1, K antigen was found to be a virulence factor [93]. Colibacillosis frequently involves serogroups O1:K1, O2:K1, and O78:K80 [94]. The serotype O86:K61 has not yet been isolated from commercial poultry. This illness has only been linked to diarrheal sickness in horses, pigs, and calves [95]. It has also been linked to cellulitis in broiler chickens. The human *E. coli* clonal group that was recovered from cases of newborn meningitis, urinary tract infections, and septicemia included the APEC serotypes O1:K1, O2:K1, and O18:K1, which are potentially zoonotic strains [96]. Humans and animals can contract the UroPathogenic *E. coli* strain serogroup O25b: H4-ST131 (sequencing type 131), which was recently found in chickens [97].

### Enzyme-linked immunosorbent assay (ELISA)

ELISA is widely used in veterinary medicine as a diagnostic tool and quality control measure to detect specific pathogens like *E. coli* in food products [98]. For research, this technique is also employed as an official veterinary diagnostic test to identify particular antigens or antibodies in production animals (such as those associated with Avian Influenza, Newcastle disease, Aujeszky’s disease, and classical swine fever) [99].

Many commercial ELISA kits are capable of detecting *E. coli*. Some of them detect it in food products: SafePath^®^ *E. coli* O:157 Microwell ELISA^®^ (SafePath^®^, USA), REAGEN™ *E. coli* O157:H7 Elisa Test Kit (REAGEN™, USA), and 3M™ TECRA™ *E. coli* O:157 (3M™ TECRA™, Germany) [85, 87]. This particular anti-*E. coli* O:157 antibody is used in a sandwich ELISA test [96]. Abnova^®^ Laboratories has a double antibody (sandwich) ELISA that uses the anti-*E. coli* O:157 antibody; BIO K 345 *E. coli* F5 ELISA Kit^®^ from Bio-X Diagnostics^®^ detects F5 pilus antigen (K99) (Bio-X Diagnostics, Belgium) antibodies from *E. coli*; and DAI^®^ *E. coli* O:157 captures the antigen from stool samples [85]. The sandwich enzyme immunoassay method known as the *E. coli* protein (*E. coli* P) ELISA Kit^®^ from MyBiosource^®^ (MyBiosource^®^, USA) is used to measure the amount of *E. coli* P in serum, plasma, and tissue homogenates [100].

### Molecular detection

The expansion of molecular biology tools to evaluate the genetic variability of many strains of bacteria, such as *E. coli*, occurred due to the discovery that prokaryotic genomes contain repetitive sequences: Repetitive extragenic palindrome, palindromic unit sequences, and enterobacterial repetitive intergenic consensus sequences [101]. The PCR reaction produces an amplified band pattern unique to each strain; subsequent use of particular primers that are homologous to this region [102].

The chuA, yjaA, and an unidentified DNA fragment known as TSPE4.C2 were detected using a quick triplex PCR technique, which was used to identify the phylogenetic group of *E. coli* strains [103]. This technique yielded 230 strains previously collected using other reference methods.

APEC research has involved the use of numerous molecular typing techniques; however, none of these techniques have identified specific genotypes. A multiplex PCR panel that targets the plasmid-borne genes iutA, hlyF, iss, iroN, and ompT, which are linked to extremely dangerous APEC strains [104]. Multiplex PCR was used to amplify 994 avian *E. coli* samples for these five genes [104]. These five genes allowed PCR to distinguish APEC strains from *E. coli* isolates from bird feces [105].

## Pathophysiology

### Omphalitis and yolk sac infection

One of the most frequent reasons why chickens die in their first few days of life is omphalitis, or inflammation of the navel [106]. The bacteria most commonly linked to these deaths are *E. coli*. Eggs are more vulnerable to fecal contamination. Nevertheless, germs can enter the bloodstream through the intestines. For example, *E. coli* infections can enter through an unhealed navel and contaminate the egg yolk sac [107]. Clinical indicators of omphalitis include edema, erythema, hardness of the skin around the navel, yolk sac, and swelling [108].

### Cellulitis

This illness is known as cellulitis and is caused by subcutaneous inflammation in the thighs and lower abdomen [109]. *E. coli* is the pathogen responsible for this condition. Cellulitis in chickens does not show any overt symptoms, and it is only observed in slaughterhouses, where it spreads to chickens kept in cages [110]. The condition is typified by superficial skin lesions arising from bird and bedding that come into close contact [111]. Cellulitis-related lesions in hens are believed to be caused by a compromised innate immune response, especially in heterophils [112].

### Swollen head syndrome (SHS)

SHS is characterized by acute or subacute cellulitis affecting the head’s periorbital region and surrounding subcutaneous tissue [83]. The first documented case of SHS was linked to coronavirus and *E. coli* infection and reported in South Africa [113]. An inflammatory exudate produced by bacteria under the skin, generally *E. coli*, causes swelling in the head, which is followed by viral infections, such as infectious bronchitis virus and avian pneumovirus [114].

### Acute vaginitis

This condition is more common in turkey and, shortly after insemination, results in acute and deadly vaginitis [115]. The most common cause of *E. coli* infection is hymen perforation, which can lead to internal laying, protrusion of the intestines and digestive tract, peritonitis, vaginitis, and egg binding [116].

### Salpingitis/peritonitis

Salpingitis affects laying hens and broiler chickens and is characterized by *E. coli*-induced oviduct inflammation [117]. Salpingitis may be linked to infections in the fallopian tubes, vents, or entire body [118]. In hatcheries, asymptomatic infections can cause decreased egg production and higher embryo mortality, even when not noticeable [119]. Peritonitis is characterized by caseous discharge and significant inflammation in the body cavity [120].

### Orchitis and epididymitis

Genital tract infections in male chickens can result in orchitis, similar to salpingitis in hens [121]. The frequency of *E. coli* infections is increasing, resulting in bigger, harder, irritated, and irregular testicles [122].

### Colisepticemia

High morbidity and mortality rates are associated with colisepticemia in laying hens and broiler chickens [35]. The prevalence of additional secondary infectious agents, such as viral infections and mycoplasma, is correlated with mortality rates [84, 123]. Approximately 80% of colibacillosis cases are associated with serotypes O2, O78, and O1, the most prevalent serotypes worldwide [124]. The primary cause of colibacillosis is high AMR, which renders antibiotic treatment useless [125]. Nevertheless, laying hens, broilers, and turkeys are susceptible to this disease because the vaccinations currently on the market do not offer sufficient immunity to protect poultry against it.

### Coligranuloma (*Hjarre’s disease*)

An uncommon type of systemic colibacillosis that can affect turkeys, laying hens, and broiler chickens is called coligranuloma [39]. Normally, this disease affects only some birds, but if it spreads to all birds, it can result in a significant fatality rate of up to 75% [74]. Granulomas are the hallmark of this condition and can be found in the liver, duodenum, cecum, and mesentery, among other organs [121]. Common lesions of coligranulomas resemble leukosis tumors [126]. Hepatic coagulation, some heterophils, and large cell counts were noted [85]. Pyogranuloma typhlitis and hepatitis in turkey are linked to coligranuloma [127].

## Transmission

### Housing conditions, ventilation, and stress

The impact of unfavorable environmental conditions has also been documented despite direct contact being demonstrated to be a significant element in transmission in APEC [128, 129]. The respiratory systems of birds are harmed by inadequate ventilation, high dust concentrations, or other chemical vapors in chicken houses [130]. Scratches or wounds in the injured respiratory tract can allow APEC to enter, leading to the development of airsacculitis, polyserositis, and potential septicemia [88]. Excessive ammonia concentrations can weaken the cilia lining the epithelium, impairing the bird’s ability to filter dust and dangerous pathogens from its respiratory system [131]. High ammonia levels are typical because ventilation is decreased in colder climates to reduce heating expenses. Reduced ventilation leads to moist air inside the poultry house, which raises the water content in the droppings and provides a perfect environment for bacteria to release large amounts of ammonia and break down uric acid [132]. This can damage the bird’s respiratory system and increase the possibility of APEC transmission.

Cleaning the poultry house while the birds are in their cages is not recommended because it can release feces-contaminated dust. Inhalation of contaminated fecal dust is a confirmed method of APEC transmission in poultry flocks [133]. Dust serves as a place for *E. coli* to grow. For this reason, *E. coli* has been identified in livestock dust that has been kept for up to 35 years at 4°C [134]. Furthermore, a preliminary investigation on poultry farms found that most poultry houses had a lot of dust on the walls and windows, which could raise the risk of APEC transmission in the bird house [16]. According to additional research, dust in and around chicken buildings is believed to be a significant factor in the spread of APEC [135]. Therefore, well-ventilated poultry barns are believed to prevent the spread of APEC and other diseases in chickens.

Poor husbandry practices, high chicken densities, and the advent of sexual maturity have all been demonstrated to stress chickens, which raises the risk of infection [132]. Increased housing density has also been linked to increased rates of germs, including *Salmonella* Enteritidis, in the intestines of chickens [136]. The same might also hold true for APEC transmission, although more investigation is necessary because various bacteria might have distinct growth needs.

### Contaminated water, feed, and eggs

Water could play a significant role in APEC’s dissemination to poultry. Pathogenic *E. coli* serotypes can be introduced into chicken flocks through contaminated well water, leading to APEC transmission [2]. The spread of APEC can be assisted by urban chicken farms that use recycled wastewater [137]. *E. coli* bacteria isolated from final effluent released from two wastewater treatment plants in the Eastern Cape Province, South Africa, were multidrug-resistant [138]. If water is used for chicken rearing, the final effluent discharge may represent an equally significant risk [139]. There is a possibility that contamination of feed and feed ingredients could result in the emergence of novel disease strains. In addition, there is evidence that the type of food fed to hens affects their intestinal microbiota [140]. It has been demonstrated that some food ingredients encourage the growth of specific gastrointestinal bacteria while inhibiting the growth of other bacteria.

Feces-contaminated eggs can cause *E. coli* infection in the yolk sac during hatching, which is typically linked to high rates of chick mortality [141]. Poultry can transmit typhus and pullorum from one generation to another through contaminated eggs [142]. Although there may be differences in the risk factors for APEC transmission between chickens and typhoid and pullorum, it is believed that tainted eggs may increase the likelihood of APEC transmission [143]. Reduced incidence of avian colibacillosis has been demonstrated by fumigating eggs within 2 h after laying and discarding eggs with cracked shells or stained by feces [74].

### Underlying chicken disease

Colibacillosis frequently coexists with other illnesses, such as Mycoplasma and respiratory virus infections, making diagnosis and treatment challenging for farmers [1]. Conversely, healthy hens have a strong immune system and are naturally resistant to *E. coli*, which is present naturally [144]. Livestock can be more vulnerable to APEC infections if they have immunity abnormalities brought on by acute infections, specifically infectious bursal disease, adenovirus Newcastle disease, reovirus, Marek’s disease, and infectious bronchitis [145]. Furthermore, the role of underlying illnesses and serum antibodies against viruses such as Newcastle disease and infectious bronchitis virus in the spread of APEC are currently being studied.

### Vectors of disease and cannibalism

Both domesticated and farmed poultry can transmit APECs to other animals. Surveys have revealed that prey birds frequently enter chicken homes, raising the possibility of infection transmission, including APEC [12]. Rodents, flies, chicken mites, foreign objects, and rodents can also serve as APEC vectors in addition to humans [146]. It has been shown that bacteria identical to those found in animal waste and multidrug-resistant clonal lineages can be transferred to different substrates by flies and other insects [147]. According to reports, flies gathered in poultry barns contained *E. coli* that produce extended-spectrum beta-lactamase, which raises the possibility of APEC transmission even more [148].

In addition, rodent droppings may be the primary cause of APEC. Antibiotic-resistance genes can be transferred between bacterial strains by rodent droppings [146]. Furthermore, it has been demonstrated that flea infestations in poultry flocks stress chickens. Therefore, it has been hypothesized that flies can transfer APEC from lesions in sick hens to healthy chickens and that fleas or other parasites feed on the blood of chickens [16]. In this study, insect control was suggested as a crucial herd management strategy to lower the spread of APEC. In addition, it was discovered that cannibalism and perking wounds are two ways in which APEC spreads among chickens [26].

### Proximity to other animals, poultry farming, and poultry density

The risk factor for the transmission of poultry diseases is the distance between poultry farms. Reducing the number of chicken farms in a region and the number of chickens on each farm is one of the most effective ways to prevent colibacillosis [142]. If biosecurity regulations are not strictly implemented, backyard flocks of chickens that usually coexist with wild birds should be separated from commercial chickens [149]. It was reported that between 2002 and 2003, private flocks in the United States experienced an outbreak of exotic Newcastle disease, which later spread to commercial chicken flocks [150]. Backyard flocks can spread zoonoses and other highly contagious infectious diseases to commercial poultry and are frequently exposed to avian influenza. The possibility of APEC transmission in poultry is thought to be increased by interactions between maintained birds and other chicken species, particularly backyard flocks. However, APEC transmission differs from that of avian influenza and Newcastle disease viruses [151].

### Vertical transmission

*E. coli* oviduct infection is a common cause of mortality in laying hens and broilers and egg production. Vertical transmission has previously been demonstrated as a means by which bacteria with AMR genes can spread [152]. Vertical transmission has also been demonstrated in other bacterial species, such as *Salmonella enterica* and *Enterococcus faecalis* [153]. For the 1^st^ time, Giovanardi *et al*. [154] documented vertical transmission of APECs from parent to offspring; previously, *E. coli* studies only examined outbreaks.

Peterson *et al*. [155] and Bortolaia *et al*. [152] later reported the transmission of *E. coli* resistant to fluoroquinolones, nalidixic acid, and ampicillin from parent to broiler. Vertical transmission of APEC has been documented by isolating *E. coli* clones from salpingitis-peritonitis lesions in broiler broodstock [156]. The transmission of *E. coli* to day-old chicks is linked to increased risk [157].

## Vaccination

Chickens are immunized with various vaccines to prevent colibacillosis. Vaccines are available in subunit, recombinant, inactivated, and live attenuated forms. Nevertheless, commercially available vaccinations do not provide sufficient immunity to protect birds from APEC strains [158].

Numerous efforts are being made to identify the best and most immunogenic *E. coli* vaccination strategies for chickens. A previous by Qiu *et al*. [159] employed bacterins; currently, recombinant or subunit vaccinations are popular. The primary issue here is that this vaccine can immunize birds in three different ways, regardless of whether it is a recombinant, attenuated, inactivated, or subunit vaccine. In summary, the vaccine should provide cross-immunity against several serotypes present in APEC. This can be achieved using various delivery methods, including food, drink, *in ovo*, and spraying [160]. This technique enables the widespread use of vaccination vaccines in chicken houses. Not less significant than the previously listed elements, the vaccine for APEC must immunize broiler chickens against APEC strains by the time they are 21 days old, as this is the critical period at which the birds can become infected [161].

Studies have demonstrated the benefits of vaccine use [158, 159]. Live vaccines in broilers reduce antibiotic use. Spray vaccination of day-old broilers against *E. coli* reduced the number of *E. coli* isolates from internal organs in the 6^th^ week of life. *E. coli* isolates from vaccinated birds were more susceptible to antimicrobials. Vaccination of broilers against *E. coli* should be considered in routine immunoprophylaxis [162, 163]. Autogenous *E. coli* vaccines are widely used in the field to prevent *E. coli* peritonitis syndrome in laying hens. Based on the results of the study, groups of laying hens that had been vaccinated intramuscularly at 14–18 weeks of age with an inactivated vaccine formulated either as an aqueous suspension or as a water-in-oil emulsion were challenged homologously or heterologously by aerosol at 30 weeks of age. Vaccination has been shown to have no effect on body growth, and both types of vaccines induced (almost) complete protection against homologous challenge [164].

## Control

Hatching egg contamination is one of the main ways that APECs spread throughout chicken flocks [165]. Frequent precautions should be taken to prevent contamination. To maintain the cleanliness of nest materials, it is necessary to regularly gather eggs, dispose of eggs that are contaminated with dust and excrement on the ground, and disinfect or fumigate eggs as soon as they are laid, ideally within 2 h [36]. These actions assist in lowering APEC transmission. APEC can be decreased or eliminated by sanitizing shell surfaces [158]. Cleaning agents work better when applied by electrostatic spraying [166]. Irradiation with UV light is another efficient strategy that can minimize or remove APEC and other infections while not interfering with incubation or affecting the hatchability of eggs [158]. Care and handling of the contaminated eggs must be performed as closely as possible during the incubation and hatching phases, as broken eggs could spread the infection to other chicks.

There is also a possibility that the hatchery’s equipment and handling staff could contaminate other chicks [2]. The vulnerable stage of the egg is until it hatches. During incubation and hatching, several recommendations for prevention and dissemination strategies exist. By opening the incubator to the outside air and, if possible, using various configurations, cross-contamination and losses can be reduced [167]. Hatcheries can contaminate people who are exposed to APEC on farms or in other hazardous areas [20]. The chicks that could be exposed to APEC should be fed frequently and kept warm.

A balanced meal high in selenium, protein, and Vitamins A and E generally increases the probability of survival in poultry [168]. However, since selenium inhibits antibody formation and causes cellulitis and colibacillosis, excessive consumption of this mineral in food can also be harmful [169]. The nutritional value of chickens is directly correlated with the severity of colibacillosis. It has been demonstrated that a feeding regimen based on alternating days is more effective for curing APEC infections in birds than regular feeding [170].

Various aspects need to be taken into account to lower the rate of APEC transmission in the digestive tract and feces: Rat droppings are a source of APEC pathogen transmission, contaminated water might contain APEC, and pelleted feed has less APEC than ground feed [3]. APEC is removed by heat during the pelletization process. Another attempt to eradicate APEC infections involves supplementing feed with 5%–10% egg yolk powder [85]. Contamination can come from water sources. It is recommended that chlorination systems and nipple irrigation be employed to lessen the transmission of APEC through water [9]. These steps lower the incidence of airsacculitis and colibacillosis. Another strategy for eliminating APEC strains from chick intestines is competitive exclusion. It has been reported that several competitive exclusion techniques, such as the use of *Bacillus subtilis* spores or commercial competitive exclusion products, provide resistance to chick microflora [171].

Maintaining adequate air quality and bedding is recommended to prevent colibacillosis from infecting the flock [172]. Proper cage ventilation reduces the amount of bacteria exposed while maintaining low levels of dust and ammonia [36]. Due to the high concentrations of dust and ammonia in the poultry environment, APEC can attach itself to these particles and be swallowed by chickens, leading to respiratory system infections [173].

APECs can endure and proliferate in moist waste. To minimize the moisture content of litter, approximately 100 feet/min of proper air velocity is required. At this rate, the garbage stays dry, lessening APEC growth [16]. Farmers should use caution when irrigation because water leaks can dampen litter and alter the conditions in which APEC breeds [2]. Farmers also need to be mindful of storing discarded materials near the manure-watering area, removing them when necessary, and replacing wet materials with dry materials when necessary.

APEC infections in poultry are frequently managed with antibiotics [174]. Numerous antibiotics from various classes, including tetracyclines (tetracycline, oxytetracycline, and chlortetracycline), sulfonamides (sulfadimethoxine, trimethoprim, sulfadiazine, sulfamethazine, sulfaquinoxaline, and ormethoprim), aminoglycosides (apramycin, gentamicin, neomycin, and spectinomycin), penicillins (amoxicillin and ampicillin), cephalosporins (ceftiofur), quinolones (danofloxacin, sarafloxacin, and enrofloxacin), polymyxins (colistin), chloramphenicols (florfenicol), macrolides (erythromycin), and lincosamide (lincomycin), have all been used in the poultry industry globally to control APEC infections. However, APEC is resistant to several antibiotics, suggesting that their use will be difficult in the future [16, 175–179].

## Conclusion

The most prevalent bacterial infection of chickens, APEC, is causing the global poultry sector to suffer significant financial losses. Animal health can be improved by efficient APEC control. Poultry systemic infection is caused by the coordinated action of several APEC virulence and pathogenesis factors. Given the serious problem of antibiotic resistance and the increasing potential for human infection by bacteria and genes resistant to antibiotics; it is necessary to develop antibacterials. The creation of antibacterials exclusively for veterinary use that do not exhibit cross-resistance to existing antibiotics may offer a future solution to the serious issue of antibiotic resistance and the high risk of the spread of bacteria and genes resistant to these antibiotics. In addition, an APEC vaccine that offers cross-protection against several APEC serotypes is required. The pathophysiology and virulence mechanisms of APEC should be studied to identify potential novel vaccines.

## Authors’ Contributions

ARK, IBM, SW, and RR: Drafted the manuscript. IBM, SMY, and AW: Revised and edited the manuscript. DAA, KHPR, IF, and MKJK: Prepared and critically checked the manuscript. SCR, KAF, and AHF: Edited the references. All authors have read and approved the final manuscript.
